# Combatting biofilm formation of *Klebsiella pneumoniae* and *Bacillus subtilis* clinical strains from the oral cavity using biogenic Se-NPs: molecular docking simulation and cytotoxic effects on HepG2 cancer cells

**DOI:** 10.1186/s12866-025-04142-w

**Published:** 2025-07-21

**Authors:** Abdullah Yousef, Mohammed Abu-Elghait, Mohamed S. Rizk, Marwa Salah Abdel-Hamid, Salem S. Salem, Gamal M. El-Sherbiny

**Affiliations:** 1 Basic & Medical Sciences Department, Faculty of Dentistry, Al-Ryada University for Science and Technology, Sadat City, Egypt; 2 Department of Botany and Microbiology, Faculty of Science, Al-Azhar University, Nasr City, Cairo, 11884 Egypt; 3 Microbial Biotechnology Department, Genetic Engineering and Biotechnology Research Institute, University of Sadat City, Menofyia governorate, Sadat City, Egypt

**Keywords:** Multi-Drug-resistant Bacteria, Se-NPs, Biofilm formation, Molecular Docking, HepG2 cell line

## Abstract

Bacterial biofilms are one of the primary causes of pathogenic activity in the oral environment; they adhere to both natural and artificial oral surfaces, causing cariogenic processes that result in dental decay and significantly reducing the lifespan of dental restoratives and prostheses; they can also affect the tissues surrounding teeth, causing gingival inflammation; persistent biofilms can cause damage to the alveolar bone, which in extreme cases may result in tooth loss; our study aims to isolate clinical isolates that are resistant to multiple drugs, before disarming them by suppressing the formation of biofilms. *Klebsiella pneumoniae* A11(*K*. *pneumonia*e) and *Bacillus subtilis* A33 (*B. subtilis*) clinical isolates were determined, and the most potent clinical isolates were identified as the most virulent strains for further investigations using 16 S rDNA PCR sequencing, with accession numbers PP995146 and PP995148 respectively. Synthesized selenium nanoparticles (Se-NPs) were analyzed using FTIR Spectroscopy, UV-Vis Spectroscopy, zeta potential, dynamic light scattering (DLS), X-ray diffraction (XRD), energy-dispersive X-ray analysis (EDX) of the [Se-NPs] solution revealed that it contained 88.49% selenium and 11.51% carbon, scanning electron microscopy (SEM), and transmission electron microscopy (TEM). According to TEM images, the average size of Se-NPs was 45.4 nm, and their shape was nearly spherical. The minimum inhibitory concentration (MICs) of biogenic Se-NPs were 0.25 mg/mL for *K. pneumoniae*A11 and 0.125 mg/mL for *B. subtilis*A33, with inhibition zones of 11–14 mm. Se-NPs significantly reduced biofilm formation at 0.125 and 0.25 mg/mL (*p* < 0.05), by 85.08% in *K. pneumoniae* A11 and 75.45% in *B. subtilis* A33. A synergistic effect with azithromycin was observed, with fractional inhibitory concentration (FIC) values of 0.502 and 0.253, respectively. Molecular interactions showed Se-NPs forming hydrophobic contacts in *K. pneumoniae* LuxS Synthase (Asp52, Asp132; binding energy − 3.9020 kcal/mol) and *B. subtilis* AbbA (His3, Met4, Arg5; -4.2489 kcal/mol). Se-NPs had an IC_50_ of 2.12 ± 0.02 µg/mL on HepG2 cells.

## Introduction

Bacterial biofilms, which initially adhered to both natural and artificial surfaces in the mouth, are a significant contributing factor to oral infections [1]. They shorten the lifespan of dental prostheses and restorations by causing cavities to form, which results in tooth decay. Gingival inflammation may also result from these biofilms’ effects on the surrounding tissues. Persistent biofilms may even harm the alveolar bone if treatment is not received, which could lead to tooth loss [2]. Organised groups of microorganisms, including fungi, bacteria, and algae, form biofilms when they adhere to biological and non-biological surfaces to form functional layers. Microorganisms often require a moist environment to form in order to prevent dehydration. By providing protection tailored to their particular environment, biofilms help microorganisms withstand external stresses [3]. Biofilm life processes are very different from planktonic life activities. In addition to providing mechanical stability, biofilms strengthen survival during nutrient scarcity, promote synergistic interactions, and shield extracellular enzymes from displacement [4]. Instead of being straightforward microbial clusters, biofilms are complex matrix systems in which individual prokaryotes regulate gene expression by communicating through signal transduction and carry out specific functions within the biofilm to maintain the survival of the group as a whole [5]. Commonly caused by the production of carbapenemases or extended-spectrum beta-lactamases (ESBLs), the multidrug-resistant bacterium *K. pneumoniae* is known for its ability to form biofilms and develop resistance to various drugs, including beta-lactams such as cephalosporins and penicillin [6]. Being involved in both oral and systemic infections, *K. pneumoniae* interaction with the oral cavity is significant. Although it typically makes up a small percentage of the natural oral flora, it can be found in trace amounts, especially in individuals with immunocompromised or poor oral health [7]. Although it is not the predominant species, *B. subtilis* can be found in the oral cavity. Small amounts of it might exist as a component of the varied microbial community. Here’s a bit more about its job and interactions. *B. subtilis* produces the cyclic lipopeptide surfactin, which is well-known for its surface-active properties. It has been extensively studied for its antibacterial, antiviral, and anticancer effects. Through its interactions with cell membranes, surfactin affects cellular activity in various ways. This study demonstrates that human oral squamous cell carcinoma (OSCC) cells undergo selective apoptosis when exposed to surfactin [8].

The pathogenicity of *K. pneumoniae* and *B. subtilis* is linked to various virulence factors that enhance their ability to cause infections. These factors include the formation of biofilms, which provide a protective environment for the bacteria, facilitating their persistence on surfaces and resistance to host immune responses [9, 10]. Additionally, both bacteria produce surface structures such as fimbriae, which aid in adhesion to host cells, and capsular polysaccharides, which help in evading the host’s immune system by masking the bacterial surface and inhibiting phagocytosis [11]. One promising strategy for developing antimicrobial treatments that could potentially surpass the efficacy of traditional antibiotics is the antivirulence approach [12]. Unlike conventional antibiotics, which aim to kill bacteria or inhibit their growth, antivirulence drugs focus on targeting specific bacterial virulence factors [13]. These factors are responsible for the bacteria’s ability to cause disease. Rather than attempting to eliminate the bacteria directly, antivirulence agents work to disarm the pathogen by inhibiting components such as adhesins, toxins, biofilm formation, and capsules, which are critical for the bacteria’s ability to adhere to host tissues, evade immune detection, and induce damage [14]. By neutralizing these virulence factors, antivirulence therapies may reduce the bacteria’s pathogenicity, making them less harmful and potentially more susceptible to the host’s immune system, without promoting resistance as rapidly as traditional antibiotics [15]. There are now a lot of clinical uses for nanotechnology. A crucial component of cutting-edge medical devices, metal nanoparticles (MNPs) have unique antibacterial properties [16–18]. Commercial antimicrobial medications including antibiotics, antifungals, and antivirals may benefit from MNPs in a number of ways, including overcoming the drawbacks of their individual use [19–23]. Since nanoparticles are toxic to microorganisms, they may be able to eradicate pathogens that are resistant to numerous medications [24, 25]. Nanoparticles can be made using a variety of methods, including chemical and physical ones [26]. Later, it was found that plant molecules could significantly more efficiently create nanoparticles by carrying out the same reduction activities [27, 28]. Recently, metal nanoparticles (NPs) have found widespread use in various pharmacological and medical applications due to their exceptional properties [29, 30]. As a result, ongoing efforts are being made to develop inhibitors targeting bacterial virulence and biofilm formation. Selenium nanoparticles (Se-NPs), a nanometal of significant medical interest, have been extensively studied for their antibacterial and antibiofilm activities against pathogenic microorganisms [31]. A common strategy to enhance the effectiveness of antibiotics and make antibiotic-resistant bacterial strains more vulnerable is the use of antibiotic combinations with non-antibiotic substances, also known as antibiotic adjuvants. It has been found that microorganisms can produce (Se-NPs), which exhibit remarkable antibacterial activity against bacterial infections. The growing prevalence of multi-drug-resistant bacteria is making the treatment of bacterial infections with traditional antibiotics increasingly difficult [32]. Se-NPs exhibit anticancer properties in certain cell lines such as PC-3 (prostate cancer cells) and LNCaP (androgen-dependent prostate cancer cells). Stabilizing (Se-NPs) with bacterial membrane components not only stops them from aggregating, but it also improves their dispersity, increases their bioavailability, decreases their toxicity, encourages cell uptake, and makes them suitable for use in medicine. Se-NPs were found to be selective against cancer cells and inert on healthy ones. These features hold promise for targeted therapy, since most chemotherapeutic treatments damage both normal and germ cells [33]. Our hypothesis is based on the disarmament of bacteria through inhibition the pathogenicity of multi-drug resistant bacteria. This approach aims to enhance immune function and reduce the severity of infections caused by *K. pneumoniae* and *B.subtilis*. By targeting biofilms as key virulence factors in these strains, Se-NPs are investigated in the study as a possible anticancer and therapeutic agent.

## Materials and methods

### Test organisms

Clinical isolates of *B. subtilis* A33 and *K. pneumoniae* A11 were utilized to assess the antibacterial and anti-virulence properties of Se-NPs. According to the Clinical and Laboratory Standards Institute (CLSI) standards, these isolates were identified as MDR bacteria and previously described as the most effective strains for biofilm formation [34]. 16 S rRNA gene sequencing analysis was used to genetically identify each isolate [35]. The National Center for Biotechnology Information (NCBI) received clinical isolates of *K. pneumoniae* A11 and *B. subtilis* A33, which were assigned accession numbers PP995146 and PP995148, respectively.

### Biosynthesis and characterization of Se-NPs

#### Sample collection

Citrus limon was purchased at the local fruit market in Egypt, whereas sodium selenite (Na_2_SeO_3_), a metal salt, was purchased from Sigma Aldrich distributors.

#### Limon peel extract preparation

To make fresh peel extract, the citrus limon was first peeled, and then the peels were sliced into little pieces with a sterile knife. After mixing about 70 g of the fresh peels with 300 mL of double-distilled water, the mixture was brought to a boil in a water bath for ten to twenty minutes. Following boiling, a series of Whatman filter sheets with varying particle sizes were used to filter the peel extract. The filtrate was then stored at 4 °C in labeled, sterile glass jars [36].

#### Sodium selenite (Na_2_SeO_3_) salt solution preparation

First, 50 mL of sodium selenite solution was used to create a 1 M stock solution, and then dilution of 10 mM was made from that stock solution to be employed in the manufacture of nanoparticles.

### Synthesis of Se-NPs

A 10 mM Na_2_SeO_3_ solution and fresh citrus limonin peel extract were mixed in a beaker in a 2:1 ratio (2 parts fruit extracts and 1 part sodium selenite solution). A magnetic stirrer was then used to agitate the liquid for 25 to 30 min in order to create a homogenous mixture. The pH, or neutral, was set at 7. After that, it was placed in an orbital shaker at 200 rpm and 70 °C for three hours in the dark. After three hours, there was a slight hue shift in all of the sample mixes, and over the next 72 h, the temperature was reduced to 37 °C. All of the solutions’ colors drastically altered after three days, going from pale yellow to brick red. After that, the sample tubes were moved from the shaker to the incubator, which maintained a steady temperature. The nanoparticles settled in the falcon tube’s base after two or three more days of incubation.

### Characterization of Se-NPs

Se-NPs were characterized using a variety of methods, including visual inspection. To identify Se-NPs, Shimadzu UV-visible (UV-vis) spectroscopy was employed. Fourier transform infrared (FTIR) spectroscopy was used to analyze the surface chemistry of Se-NPs and biomolecules in a solution of extract from limon peels. Zeta potential measurements and dynamic light scattering (DLS) were used to assess the surface and colloidal stability of Se-NPs. X-ray diffraction analysis (XRD) is used to ascertain a material’s crystallographic structure. The Se-NPs surface shape and particle size were assessed using transmission electron microscopy (TEM). The energy dispersive X-ray analysis (EDX) approach, which is commonly employed in contemporary scanning electron microscopy (SEM), uses the activation of nanoparticles to ascertain the elemental composition or chemical characterization of a material.

#### UV-vis spectral analysis

Typically, 300–800 nm wavelengths are employed to characterize metal nanoparticles in the 2–100 nm size range. UV-vis spectral analysis checks the stability of metallic nanoparticles.

#### Zeta potential & dynamic light scattering

Charge was measured using a zeta potential analyzer, while the aggregation kinetics measured using DLS to clarify the adsorption mechanism of a synthetic nanoparticles.

#### FTIR studies

Before and after the bio-reduction process, there are notable differences in the characteristics of the leaf extract. The FTIR investigations demonstrate that proteins with amide groups are better at binding metal ions, suggesting that the protein form layer encased the metal nanoparticles.

#### XRD analysis

An approach in materials research that establishes a material’s crystallographic structure [37].

#### TEM analysis

In Transmission Electron Microscopy (TEM), a high-energy electron beam with a consistent current density, typically ranging from 60 to 150 keV, is directed toward a thin sample. Upon interaction with the material, the beam undergoes two main types of scattering: elastic scattering, where electrons are deflected without energy loss, and inelastic scattering, where electrons lose energy due to interactions with the sample, such as exciting inner-shell electrons or inducing vibrations in the atomic lattice. Some of the electron beam also transmits through the sample, providing information about the internal structure of the material. The degree of scattering and transmission depends on various factors, including the thickness of the sample, its density, and the elemental composition of the material, which determine how the electron beam interacts with the atoms of the sample.

#### SEM and EDX analysis

The present study uses EDS/EDAX to determine the composition of minerals in grains. EDS/EDAX is depicted as a characteristic (in keV) in a plot of X-ray. The study has examined many elements of the prenet in this standard EDS/EDAX. The majority of elements produce several peaks, but they are narrow and easy to control. Generally speaking, particles smaller than 0.2 mm cannot be observed or measured with a standard handheld microscope. The SEM displays details about the sample, including its location, crystalline structure, chemical composition, and peripheral morphology.

### Determination of minimum inhibitory concentration (MIC) of Se-NPs against the most potent clinical strains

For the most powerful bacterial strains, the minimum inhibitory concentration (MIC) of the test substance Se-NPs was estimated using the broth microdilution method. In summary, nutrient broth was used to prepare the overnight-grown culture. For every bacterial strain in the microtiter plate wells, the inocula 0.5 Mc Farland 1.5 × 10^8^ CFU was modified to reach a cell concentration of 10^8^ CFU/mL. The ultimate capacity of each well was 200 µl, which included 180 µl of bacterial culture and 20 µl of Se-NPs in different doses. The growing medium devoid of inoculum and well-containing suspended Se-NPs was used as the negative control, while the organism devoid of Se-NPs was used as the positive control. Three runs of the experiments were conducted, and the mean results are shown. In accordance with CLSI criteria, the test was conducted and the results evaluated. After the incubation period, the cell density at O.D. 620 nm was measured using a microplate reader (STATFAX, USA). The MIC of Se-NPs that produced no turbidity or bacterial growth in comparison to the control.

### Effect of Se-NPs on biofilm formation

Two clinical strains that were most successful in preventing the formation of biofilms *K. pneumoniae* A11 and *B. subtilis* A33 were used to evaluate clove oil’s ability to do so. Inoculation allowed the cells to form biofilms by exposing the strains to the growth media. Twofold serial dilutions of Se-NPs were prepared in a 96-well microtiter plate (MTP) with trypticase soy broth supplemented with 1% glucose (TSBGlc) at dosages lower than the MIC values. Following that, each MTP well received bacterial suspensions (5 × 10^5 CFU/mL). The impact of Se-NPs on the biofilm formation of *K. pneumoniae* A11 and *B. subtilis* A33 was evaluated using a microplate reader (STATFAX, USA) over a 24-hour incubation period at 37 °C at 620 nm O.D. Following incubation, planktonic cells were disposed of, and the wells were cleaned three times using phosphate-buffered saline (PBS, pH 7.2). The wells were dried for 30 min and then fixed with 99% methanol for 10 min. The amount of biofilm that developed in the presence of Se-NPs was measured by the researchers by applying 0.1% crystal violet to each well for 20 min. Any crystal violet that remained was disposed of after the wells were cleaned with distilled water. Following the full drying of the wells, 30% acetic acid was added.

## Molecular Docking simulation

The molecular docking simulation of eugenol was carried out using the Molecular Operating Environment (MOE) version 2019.0901. LuxS synthase from *K. pneumoniae* (PDB: 1INN) and the biofilm matrix promoter AbbA from *B. subtilis* (PDB: 2LZF) are the target receptors whose 3D structures were made available by the Protein Data Bank (https://www.rcsb.org/structure/1INN; https://www.rcsb.org/structure/2LZF; last accessed 5/2/2025). Only one chain (chain A) was chosen to create the active sites of these receptors. Chain A was chosen, and normal techniques were followed to provide an active site for the biofilm matrix promoter AbbA (PDB: 2LZF). London dG and GBVI/WSAdG were used as the rescoring methods, while Triangle Matcher was used for placement in the docking process. Forcefield was also utilized for post-placement refining. With a negative value, the docking stance had the most binding energy.

## Drug combination assay

Combining drugs is currently a cutting-edge tactic to treat a variety of microbiological illnesses. In order to combat the multidrug-resistant *K. pneumoniae* A11 and *B. subtilis* A33 clinical isolates, this work employed the checkboard approach in conjunction with the antibacterial growth of generated 1000 µg/mL Se-NPs and the antibiotic Azithromycin. The effect is considered synergistic if the fractional inhibitory-concentration index [FIC] = [MIC of antibiotic-combined/MIC of antibiotic-alone] + [MIC of tested compound-combined/MIC of the tested compound-alone] is ≤ 1 and exhibits an antagonistic effect when FIC- index > and an additive effect when 1 < FIC- index ≤ 2.

## MTT antitumor assay

To assess the cytotoxicity of Se-NPs, the MTT test was employed. The mitochondria of living cells transform the hydrophilic tetrazolium salt, which appears yellow in this assay, into the hydrophobic purple pigment formazan. The number of surviving cells is indicated by the color’s intensity. The previously described protocol was followed for performing the MTT experiment. After seeding cells into each well of a 96-well microtiter plate on the first day, they were suspended in 100 µL of the suitable medium at a density of 1.5 × 104 cells/mL and incubated for 24 h. The next day, the formulations were applied to the wells in triplicate at the appropriate amounts after being diluted in 0.5% DMSO and filtered through 0.450 μm syringe filters. Se-NPs were tested at doses ranging from 0.4 to 100 µg/mL for the HepG2 cells from Science way Co., Egypt. For a whole day, the cells were cultivated at 37 °C with 5% CO_2_. On the third day, the old medium were swapped out for fresh ones, and the cells were incubated for four hours at 37 °C with 5% CO_2_. They were then put through a typical MTT assay after this. Each sample’s absorbance at 570 nm was measured using a microplate reader after the formazan crystals were dissolved in 100 µL of 1% HCl in iso-propanol. An exponential viability curve plotted against the substance’s concentration was used to determine the inhibitory concentration at 50% (IC_50_). The formula for calculating the percentage of cell viability is (absorbance sample/control) x 100.

## Statistical analysis

The results represent the averages of three separate studies, each of which was conducted in three replicates. A one-way ANOVA model of analysis of variance (ANOVA) (α = 0.05) was used to evaluate the data in order to ascertain the significance between groups. Multiple comparisons were conducted using Tukey’s test after pairwise comparisons revealed a significant difference. A significant difference is shown by different letters, but a non-significant difference is indicated by the same letters.

## Result and discussion

### Production and characterization of selenium nanoparticles

**Fig. 1:**
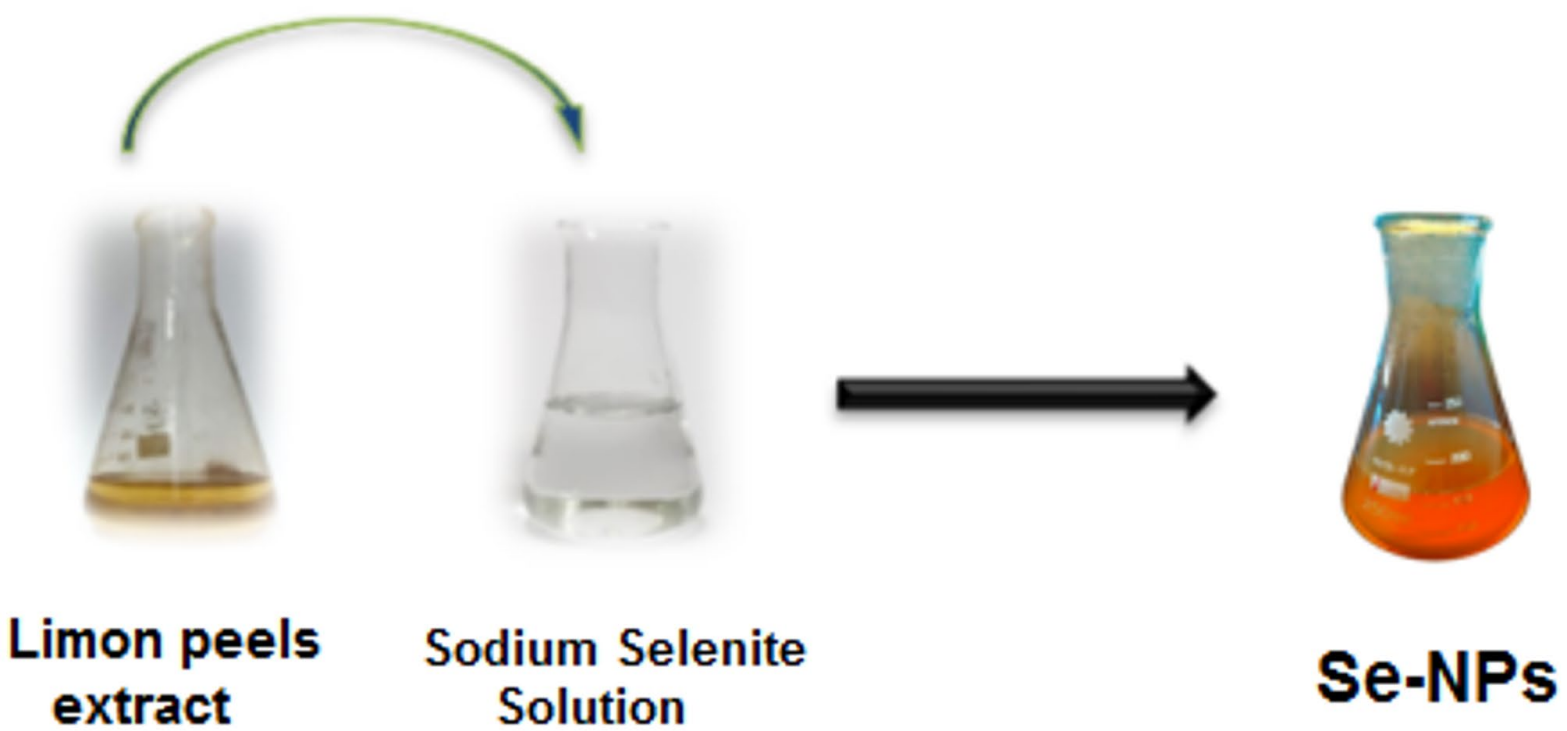
Formation of Se-NPs

Recent research has shown that lemon leaf extract is an effective medium for the green synthesis of selenium nanoparticles (Se-NPs), due to its abundance of secondary metabolites. Bioactive compounds like flavonoids, terpenoids, and polyphenols serve dual roles as reducing and stabilizing agents during the synthesis process. These molecules aid in transforming selenium ions (SeO_4_²⁻ or Se⁴⁺) into stable elemental selenium nanoparticles by providing electrons and forming a protective layer around the particles. This environmentally friendly method eliminates the need for harmful chemicals and boosts the biological functionality of Se-NPs, making them ideal for use in medical and environmental fields [38]. In current Study One hundered millileter suspension of Se-NPs with concentration 1000 µg/mL prepared by limon peels extract. Sodium selenite (Na_2_SeO_3_) solution changed from colorless to brick red after addition of Lemon leaves extract indicated the formation of Se-NPs (Figure 1).

#### UV-Vis absorption spectra

**Fig. 2:**
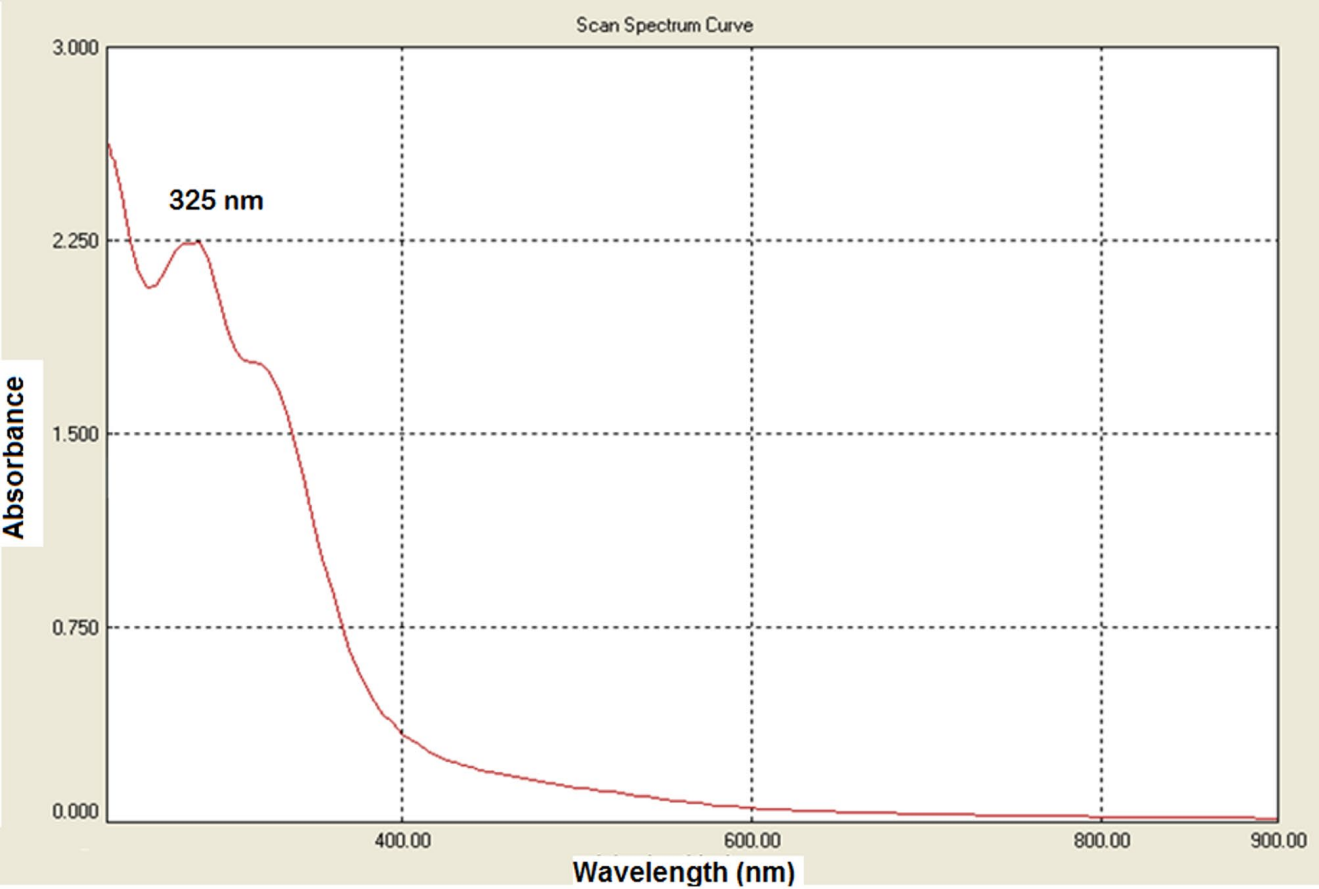
Ultraviolet-visible spectrum of Se-NPs

Based on their surface plasmon resonance (SPR), Se NPs employing *Pelargonium zonale* leaf extract had a wavelength range of 270–350 nm [39]. In current study, characterization of synthesized Se-NPs in the presence of limon peels extract was determined. Surface Plasmon absorption bands [SPR] of Na_2_SeO_3_ with limon peels extract was dropped at wavelength 325 nm as illustrated in (Figure 2) indicate the formation of Selenium nanoparticles. In the previous studies, the successful synthesis of Se-NPs was confirmed by characteristic absorption features in the UV-Vis spectrum, including a sharp peak at 275 nm and a broad peak around 390 nm, both indicative of nanoparticle formation [40, 41].

#### Zeta potential measurements

**Fig. 3:**
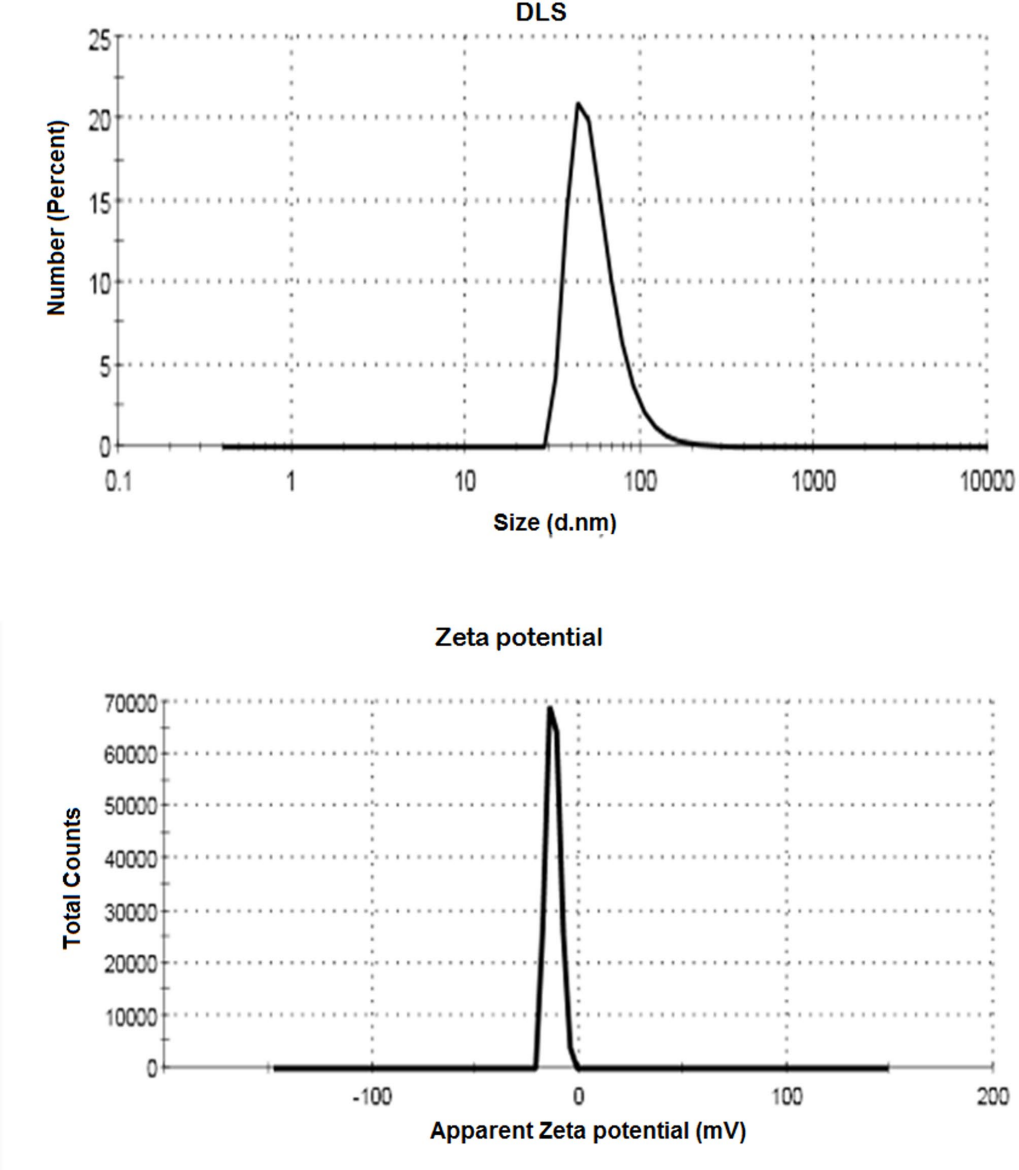
Colloidal and Surface properties of prepared Se-NPs

Using dynamic light scattering (DLS) and zeta potential studies, the surface and colloidal stability of the produced Se-NPs at various intervals were identified. It is evident that the particle size [i.e., hydrodynamic diameter, *H*_*D*_] was around 75.4 nm when limon peel extract was added to an aqueous solution of Na_2_SeO_3_, as shown in Fig. 3. In previous study, DLS analysis indicated that the biosynthesized selenium nanoparticles possessed a hydrodynamic diameter 55.9 nm, confirming effective nanoparticle formation. A polydispersity index (PDI) of 0.03 reflected a narrow size distribution, suggesting the nanoparticles were largely monodisperse with minimal aggregation. Additionally, a zeta potential value of − 17.8 mV indicated strong colloidal stability due to sufficient surface charge repulsion [42].

#### FTIR analysis

**Fig. 4:**
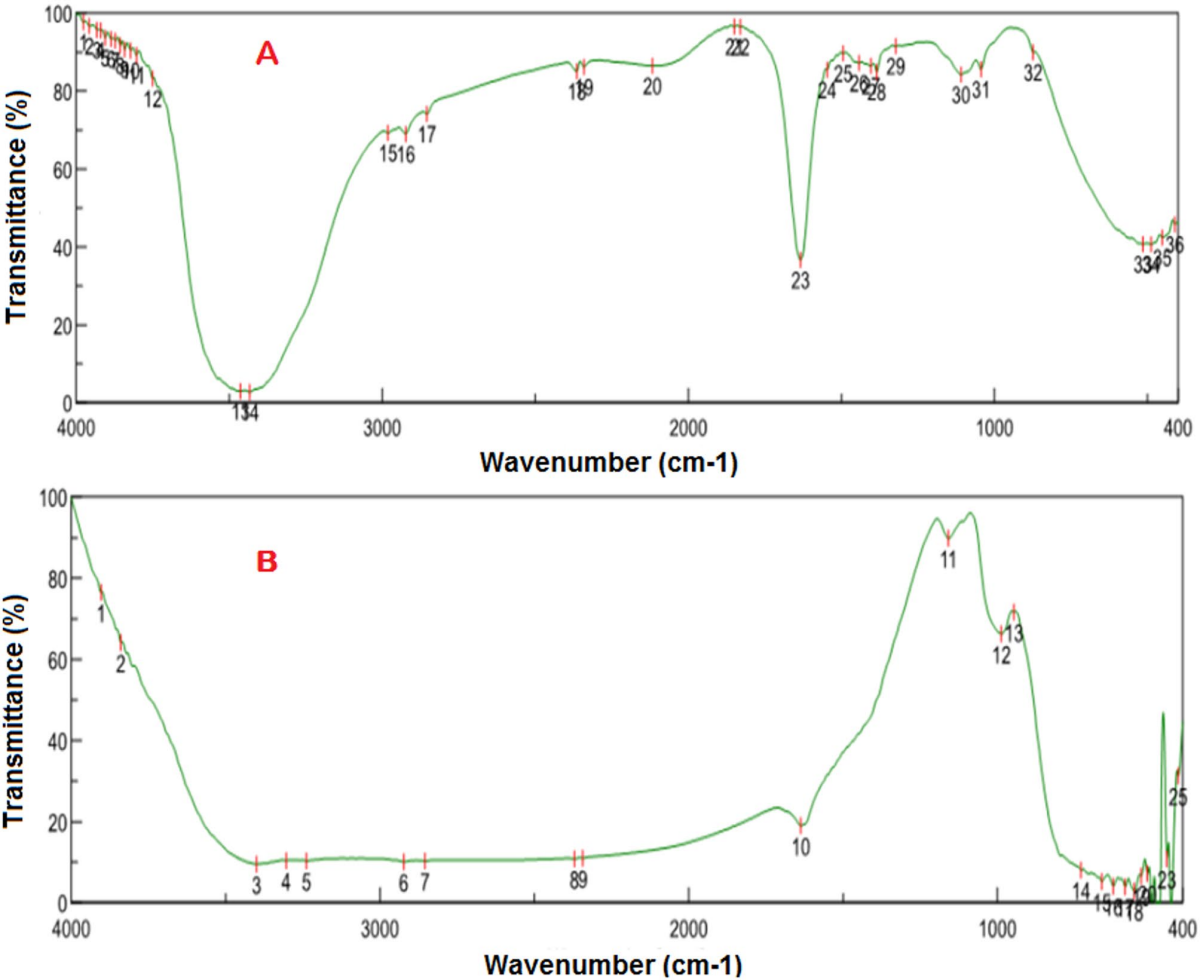
FTIR spectra analysis. **A)** FTIR spectrum of *Limon* peels extract and **B)** FTIR spectrum of Se-NPs

Our study use Fourier Transform Infrared Spectroscopy (FTIR) to identify the possible biomolecules responsible for the reduction, capping, and efficient stabilization of selenium nanoparticles. From simple organic and biological molecules to supramolecular biosystems, they have been effectively used to analyze samples of various complexity [43–45]. In the FTIR spectra of the extract from limon peels, the main peak positions are 3920, 1633, and 514 cm^-1^. The carboxylic/phenol group’s OH stretching vibrations are responsible for a peak at 3920 cm^-1^, whereas the ketone group’s C = O is linked to a broad and potent peak at 1633 cm^-1^. The peak of alkyl halides is located at 514 cm^-1^, as indicated in (Figure 4.a). The presence of so many functional groups may have contributed to the bio-reduction of Se ions. When the residual extract from limon peels is used as a capping agent, the spectra show the existence of selenium nanoparticles in the sample with a few slight changes in peak location. The presence of = C-H alkene is detected at 946.9 cm^-1^, while the alkyl amine stretching peak is situated at 1159 cm^-1^. After analyzing the band intensities for limon peel extract and selenium nanoparticles in a variety of spectral regions, many peaks from 400 to 700 finally show the presence of nanoparticles (Figure 4.b) In previous study, FTIR spectroscopy analysis revealed the involvement of various biomolecular functional groups in the green synthesis and stabilization of Se-NPs. Peaks observed at 1069 cm⁻¹ and 1252 cm⁻¹ were attributed to phenolic and other organic functional groups, suggesting their key role in the bioreduction and capping processes. Additionally, distinct absorption bands at 3266 cm⁻¹ (O–H stretching) and 2917 cm⁻¹ (C–H stretching) indicated the presence of hydroxyl and aliphatic groups, further supporting the participation of polysaccharides and other biomolecules in Se-NPs formation and stabilization [46].

#### XRD analysis

**Fig. 5:**
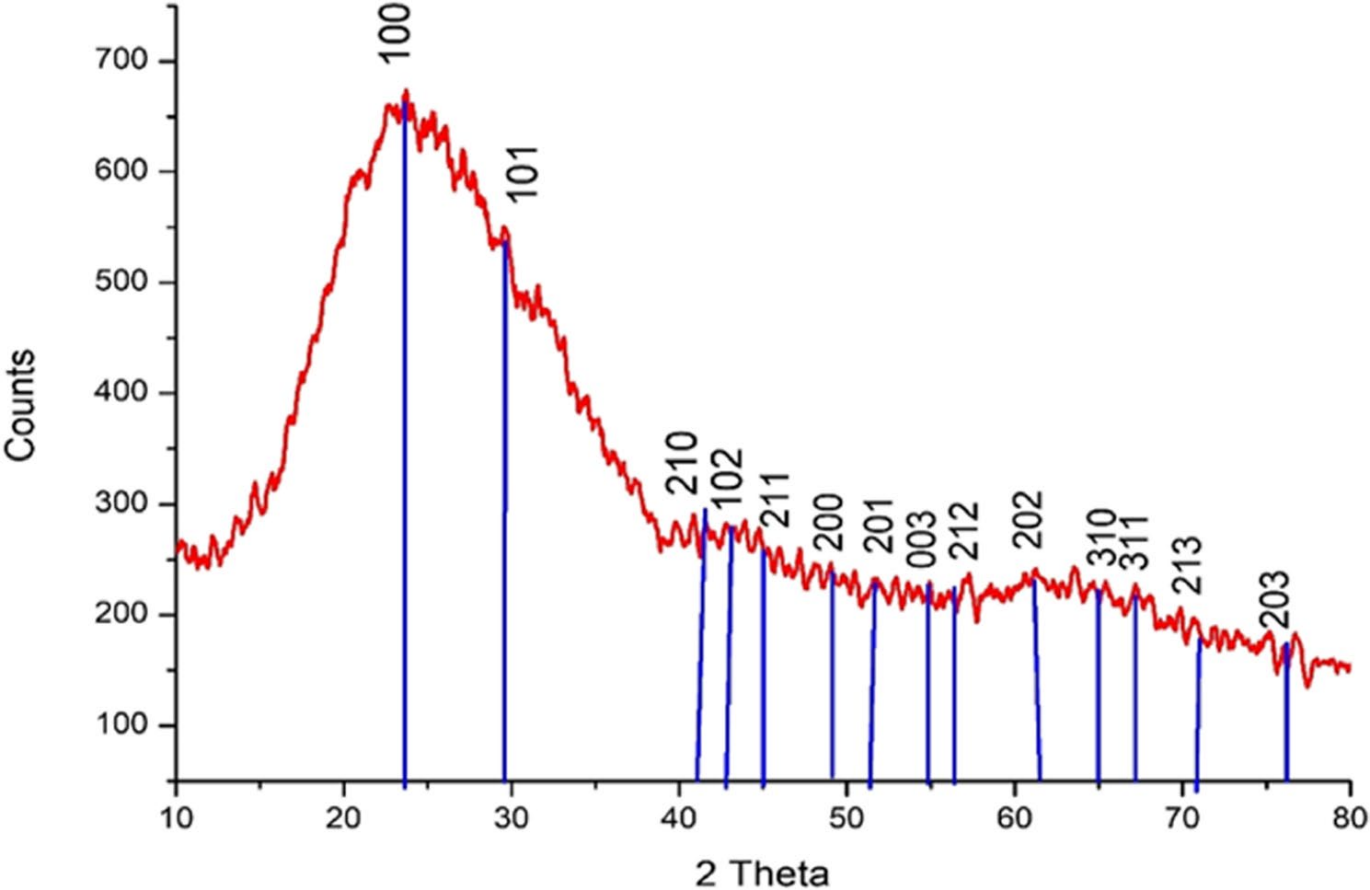
XRD Analysis of limon Se-NPs

There are weak to moderately high diffraction peaks at (100), (101), (210), (202), and the (100) plane of selenium with hexagonal lattice symmetry, as well as 20 values of 23.57°, 29.76°, 41.43°, and 61.80° in the XRD patterns of Se-NPs made using limon peel extract, as illustrated in (Fig. 5). In Previous study, X-ray diffraction (XRD) analysis of Se-NPs synthesized using *Bacillus cereus* exhibited prominent diffraction peaks at 2θ values of 23.9°, 30.0°, 41.7°, 44.0°, 45.7°, 52.0°, 56.4°, 62.2°, 65.5°, and 68.4°. These peaks correspond to the (100), (101), (110), (102), (111), (201), (003), (202), (210), and (211) planes of hexagonal-phase selenium [47].

#### **TEM** analysis

**Fig. 6:**
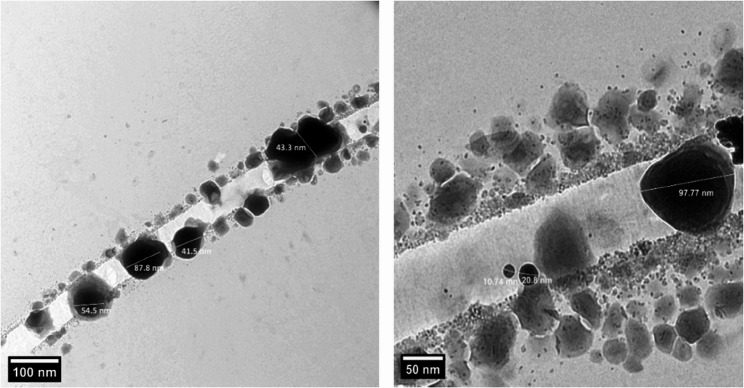
TEM micrographs for prepared Se-NPs with limon peels extract

The TEM image of Se-NPs is displayed in (Figure 6), where it was found that the nanoparticles had an almost spherical shape and an average size of 45.4 nm. The particles’ nanocrystalline structure is also shown in the pictures. In previous study, Transmission electron microscopy (TEM) analysis revealed that the Se-NPs exhibited spherical morphology and irregular shape. The particles showed a size distribution ranging from 35 to 100 nm. The observed shape diversity suggests potential implications for shape-dependent biological activity [48].

#### SEM and EDX analysis

**Fig. 7:**
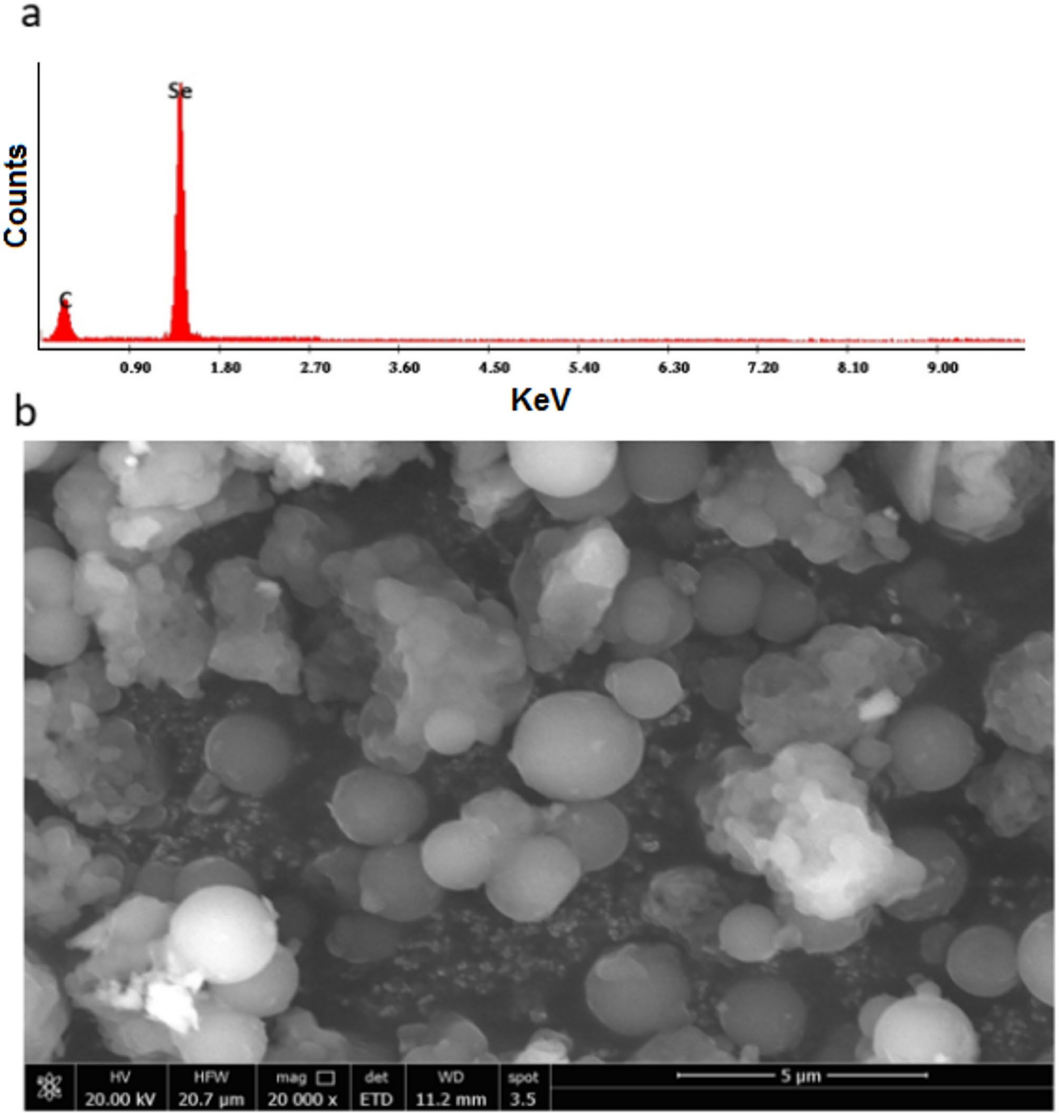
EDX (**a**) and SEM image (**b**) of Se-NPs produced by limon peels extract

In this work, the chemical composition of the biologically generated Se-NPs was confirmed by EDX analysis. (Figure 7.a) illustrates the [Se-NPs] EDX analysis. The nanoparticles contains selenium with 88.49% and carbon with 11.51%, In addition, (Figure 7.b) show SEM images of Se-NPs, the all of which are sphere shape. Se-NPs, which have an average particle size of 27 nm, exhibit the hexagonal phase according to powder XRD examination. Pure selenium peaks may be seen in the EDX spectra of the Se-NPs, however agglomerated spherical pictures with a spherical morphology are visible in SEM and TEM [49]. In previous Study, SEM analysis confirmed the spherical morphology of Se-NPs, with an average size range of 60–80 nm. EDX analysis displayed a prominent Se Lα peak at 1.37 keV, confirming the presence of selenium nanoparticles (59.7%) [50].

#### Antibacterial and MIC of Se-NPs against *K. pneumoniae*A11 and *B. subtilis*A33

**Fig. 8:**
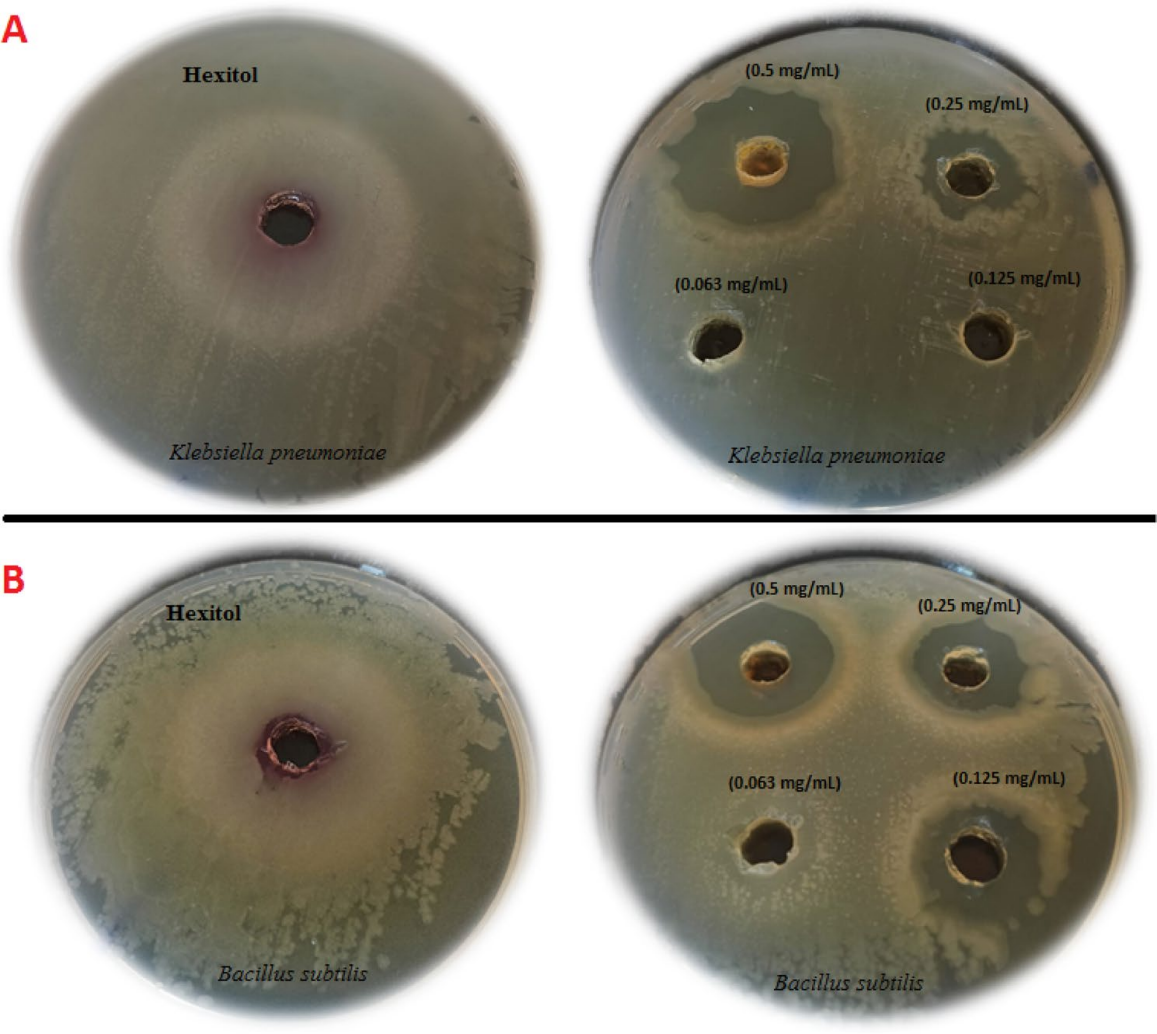
Antibacterial and MIC of Se-NPs against the most virulent clinical strains. **A**: Hexitol as appositive control and different concentrations of Se-NPs against *K. pneumoniae* A11, **B**: Hexitol as a positive control and different concentrations of Se-NPs against *B. subtilis* A33 strain

Clinical isolates of *B. subtilis*A33 and*K. pneumoniae*A11 were utilized to assess the antibacterial and anti-virulence properties of Se-NPs. These isolates were previously identified as the strains with the strongest virulence factors and classified as MDR bacteria in accordance with the recommendations of the Clinical and Laboratory Standards Institute (CLSI) [35]. Mishra et al. [51], MIC of Se-NPs made using extract from limon leaves 0.25 mg/mL as a concentration against *K. pneumoniae*. In our study, Se-NPs prepared by limon peel extract exhibit lethal activity at high concentration [1000 µg/mL] against *K. pneumoniae* A11 and *B. subtilis* A33. The minimum inhibitory concentrations (MICs) were determined to be 0.25 and 0.125 mg/mL for *K. pneumoniae* A11 and *B. subtilis* A33, respectively, with inhibition zone diameters ranging from 11 to 14 mm on agar plates. Hexitol was tested as standard antiseptic against the bacteria under study for *K. pneumoniae* A11 *and B. subtilis* A33 strains were exhibit high resistant to this antibiotic where no inhibition zone was found and MIC was > 100 µg/mL (Table 1) and (Fig. 8).

**Table 1:** Antibacterial and MIC of selenium nanoparticles against *K.pneumoniae* A11 and *B. subtilis* A33 strains

Agent	Se-NPs		Hexitol	
Strain code	Inhibition Zone (mm)	MIC (mg/mL)	Inhibition Zone (mm)	MIC (µg/mL)
*K.pneumoniae* A11	11	0.25	0	> 100
*B. subtilis* A33	14	0.125	0	> 100

### Effect of sub-inhibitory concentrations Se-NPs on biofilm activity

#### Effect of sub-inhibitory concentrations Se-NPs on biofilm activity of *K. pneumoniae* A11 strain

**Fig. 9:**
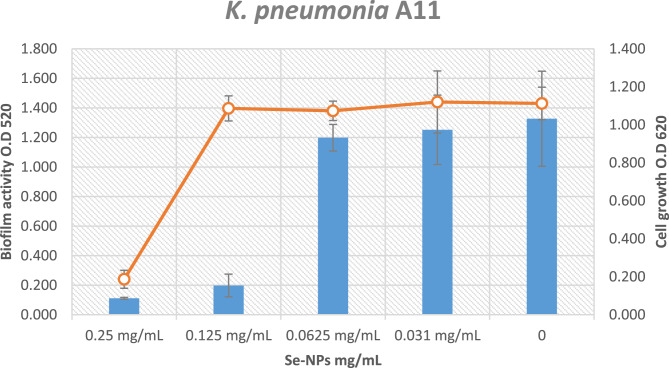
Effect of Se-NPs on Biofilm activity of *K. pneumoniae* A11strain

At a concentration of 0.125 mg/mL [*p* < 0.05], Se-NPs significantly reduce biofilm formation against *K. pneumoniae* A11 by 85.08% without influencing the development of planktonic cells (Fig. 9). Lashani et al. [52], reported that biogenic Se-NPs were tested against various bacterial strains, including *K. pneumoniae*. The results showed that a concentration of 1 mg/mL of Se-NPs inhibited biofilm formation by 51.3% in *K. pneumoniae* isolates, indicating strong antibiofilm activity.

#### Effect of sub-inhibitory concentrations Se-NPs on biofilm activity of *B. subtilis* A33

**Fig. 10:**
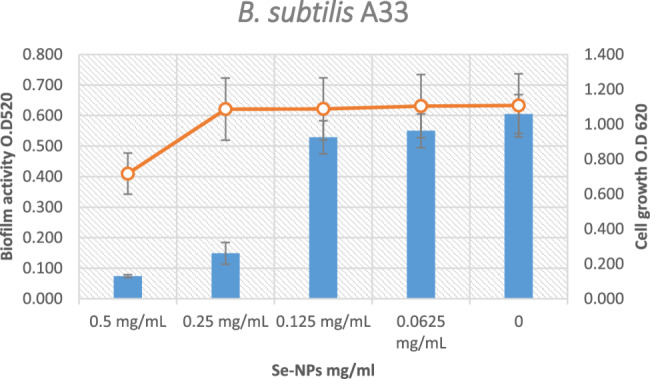
Effect of sub-inhibitory concentrations Se-NPs on Biofilm activity of *B. subtilis*A33

Without influencing the growth of planktonic cells, a concentration of 0.25 mg/mL Se-NPs significantly reduces biofilm activity [*p* < 0.05] against *B. subtilis* A33 by 75.45% (Fig. 10). Salem et al. [38], reported that the effect of Se-NPs on biofilm formation in different bacterial strains, including Bacillus subtilis. The findings showed that Se-NPs were able to reduce biofilm formation at concentrations between 0.1 and 1 mg/mL. However, the specific percentage of inhibition and the impact on planktonic growth were not provided.

## Docking simulation

**Fig. 11:**
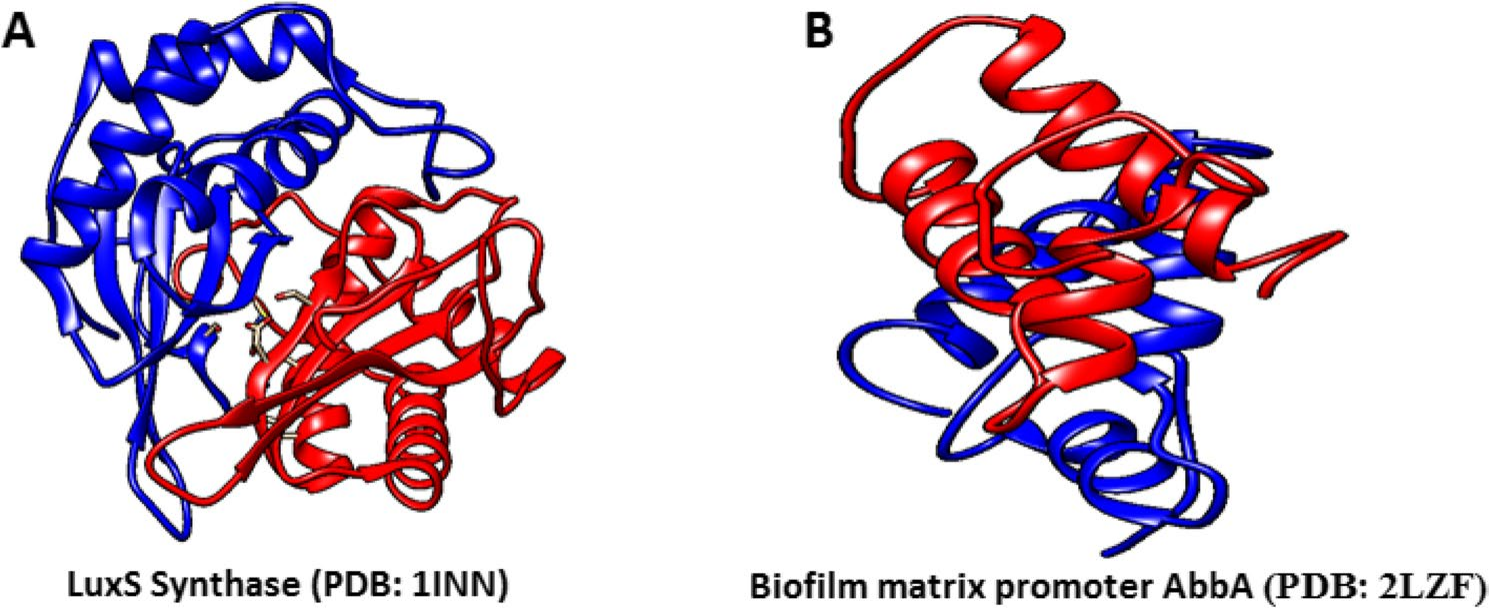
**a** 3D crystallography of LuxS Synthase in *K. pneumoniae* (PDB: 1INN), **b** 3D crystallography of the biofilm matrix promoter AbbA from *B. subtilis* obtained from protein data bank

**Fig. 12:**
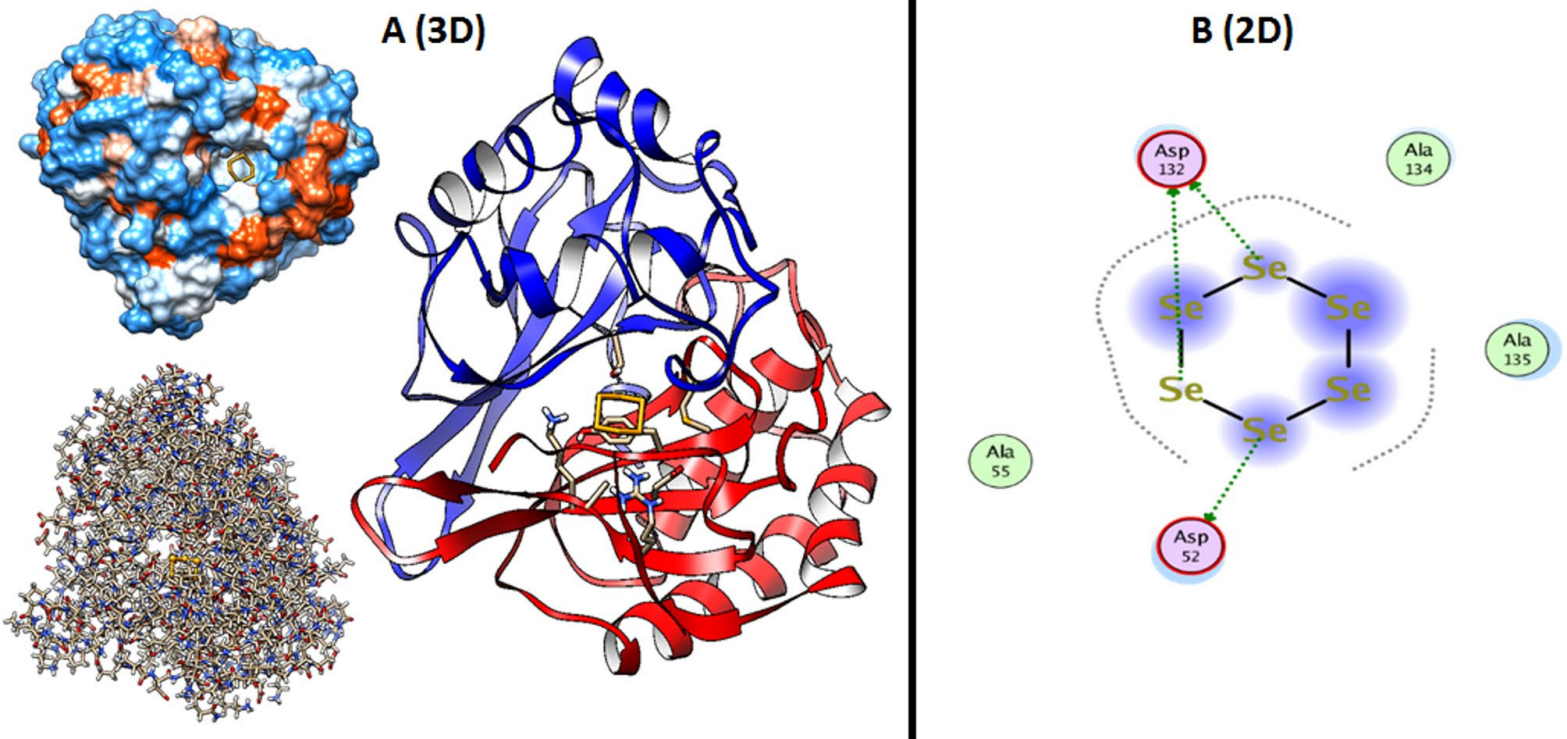
Structure of Se-NPs within the active site of the biofilm matrix promoter AbbA from *B. subtilis* (PDB: 2LZF) (**a**) 3D Structure shown the binding mode of Se-NPs inside the pocket with amino acid interaction (**b**) 2D Structure shown the binding mode of Se-NPs inside the pocket with amino acid interaction

**Fig. 13:**
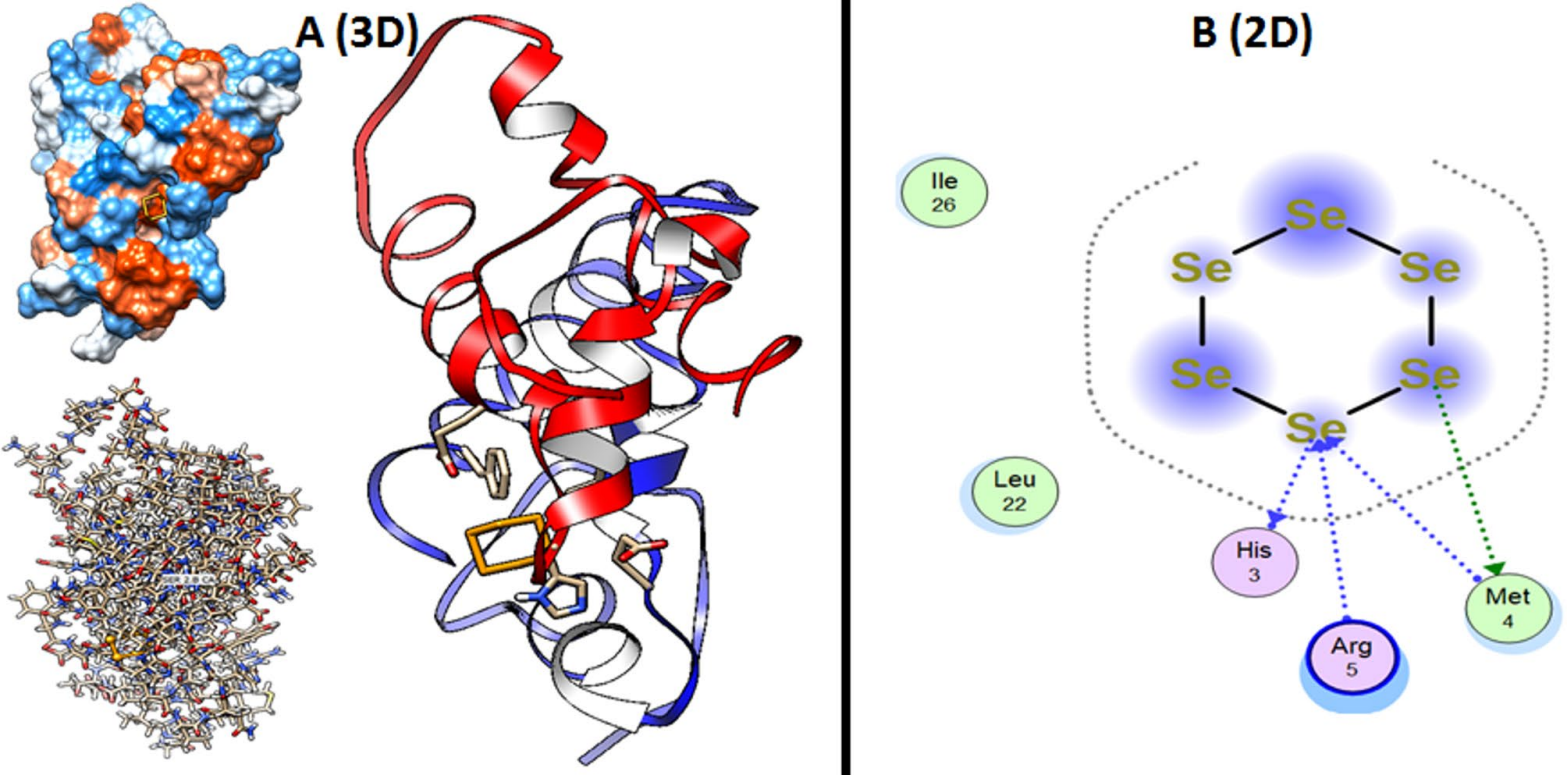
Structure of the Eugenol within the active site of LuxS synthase in *K. pneumoniae* (PDB: 1INN) (**a**) 3D Structure shown the binding mode of Se-NPs inside the pocket with amino acid interaction (**b**) 2D Structure shown the binding mode of Se-NPs inside the pocket with amino acid interaction

When utilizing computer models to anticipate the three-dimensional structure of protein-ligand complexes and comprehend their interactions, molecular docking simulation is an essential technique [53, 54]. Additionally, the docking simulation for Se-NPs can provide insights into response processes and potential pathways of action [55]. This study used docking simulation to examine the active sites of the biofilm matrix promoter AbbA from *B. subtilis* (PDB: 2LZF) and Se-NPs in the Structure of LuxS Synthase in *K. pneumoniae* (PDB: 1INN) (Figure 11). In order to discover potential targets for antibacterial action, the study set out to ascertain how Se-NPs interacted with LuxS synthase and the biofilm matrix promoter. *K. pneumoniae* LuxS Synthase (PDB: 1INN) Se-NPs demonstrated hydrophobic contact with Asp52 and Asp132 as amino acid residues inside the active site, with a binding energy S = −3.9020 kcal/mol. Furthermore, as seen in the structure of the biofilm matrix promoter AbbA from *B. subtilis* (PDB: 2LZF), Se-NPs demonstrated hydrophobic contact with His3, Met4, and Arg5 as amino acid residues inside the active site, with a binding energy S = −4.2489 kcal/mol (Figs. 12 and 13). Ultimately, we can say that the inhibition of LuxS Synthase and the biofilm matrix promoter AbbA from *B. subtilis* is how Eugenol exhibit their antibacterial effect. Yet, it can be deduced from the binding energy that Eugenol prefer LuxS Synthase and the biofilm matrix promoter AbbA more, and that their activity is demonstrated by hydrophobic interactions in the active site pocket, which is situated less than 2.12 Å away.

## Drug combination assay

The issue of multidrug resistance must be addressed in order to increase the efficacy of antibiotics [56]. They could be affixed to appropriate nanoparticles to create powerful drugs at low concentrations in order to achieve this. Neural particles of selenium Therefore, Se-NPs are considered biocompatible compounds that are utilized to prevent infections caused by bacteria that are resistant to a variety of medications [57]. Mueller-Hinton agar plates were used to investigate the medication combination of Se-NPs with the macrolide antibiotic azithromycin in order to battle clinical strains of *B. subtilis* A33 and Azithromycin-resistant *K. pneumoniae* A11. The findings demonstrated a synergistic effect, as the inhibition zone for *K. pneumoniae* A11 and *B. subtilis* A33 increased to 22 ± 0.5 and 21.5 ± 0.5 mm in diameter, respectively, when the two compounds were combined. With FIC values of 0.502 and 0.253 for *K. pneumoniae* A11 and *B. subtilis* A33, respectively. The test results showed that the combination of Se-NPs and the antibiotic azithromycin had a synergistic effect. The test was repeated to determine the MIC values for both compounds [to detect FIC value] (Tables 2 and 3).

**Table 2:** Drug combination effect of Se-NPs with Azithromycin against *K. pneumoniae* A11

MIC	Se-NPs mg/mL	Azithromycin mg/L
MIC alone	0.25	0.12
MIC combined	0.063	0.03
FIC index	Value	Effect
	0.502	Synergistic effect

**Table 3:** Drug combination effect of Se-NPs with Azithromycin against *B. subtilis* A33

MIC	Se-NPs mg/mL	Azithromycin mg/L
MIC alone	0.125	0.12
MIC combined	0.032	0.015
FIC index	Value	Effect
	0.253	Synergistic effect

### Selenium nanoparticles as antitumor agent using (MTT) assay

**Fig. 14:**
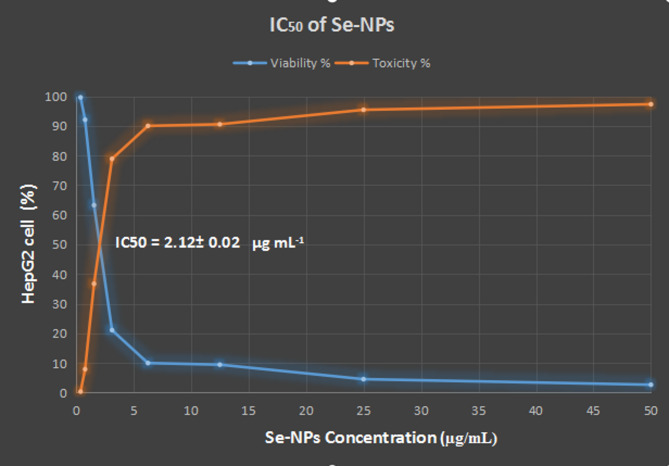
Antitumoir activity of SeNPs against HepG2 cell line

Cancer remains one of the most deadly illnesses in the modern world. It is being addressed in fresh ways every day. The use of Se-NPs has garnered a lot of attention lately because of its anticancer properties [58]. One feature that can be incorporated into nanoparticles is the ability to recognize malignant cells and enable accurate and targeted drug delivery while avoiding contact with healthy cells [59, 60]. It has been demonstrated that biocompatible, ecologically friendly nanoparticles have been created. They can improve their contact with living systems more effectively by doing this activity [61]. The detrimental impacts of nanoparticles are substantial because of their many characteristics. This category includes a number of attributes, including size, shape, concentration, and surface chemistry [62, 63]. The IC_50_ value, or the concentration of a medication required to cause 50% cell death, was calculated using the dose-response curve. The Se-NPs demonstrated an IC_50_ value of 2.12 ± 0.02 µg/mL on HepG2 cell lines, as indicated by the data in Fig. 14. Using various dosages of Se-NPs, ranging from 50 to 0.39 µg/mL, an anticancer study was conducted to evaluate the effects of Se-NPs on HepG2 cell lines. HepG2 cell viability was 99.65, 92.10, 63.26, 21.09, 9.98, 9.43, 4.54, and 2.67, in that order as shown in Fig. 14. Additionally, the distribution and movement of the nanoparticles within the malignant matrix were enhanced by their small size and surface charge [64, 65]. Further research showed that Se-NPs made with silver and chitosan nanocomposite (Se-Ag-CS NCs) had a substantial lethal effect on a liver tumor cell line [66]. In other reports, HepG2 and MCF-7 cells experienced dose-dependent cytotoxicity from Se-NPs [67–70]. In a separate study, Se-NPs were Synthesized by *Penicillium citrnium* have a significant cytotoxic effect (IC_50_) of 100.2 ± 3.28 µg/mL against HepG2 cells [71].

## Conclusion

In conclusion, our study highlights the potential of Se-NPs synthesized using lemon peel extract as a promising antimicrobial and anti-virulence agent against multidrug-resistant (MDR) clinical isolates of *K. pneumoniae* A11 and *B. subtilis* A33. The Se-NPs exhibited strong biofilm inhibition, significant antimicrobial activity, and a synergistic effect when combined with azithromycin. Structural analysis further revealed hydrophobic interactions within key active site residues of virulence-related proteins, supporting their potential as biofilm-disrupting agents. Furthermore, the Se-NPs showed a mild cytotoxic effect on HepG2 cell line, indicating that additional in vivo research is necessary to evaluate their therapeutic suitability. These results open the door to the creation of innovative nanoparticle-based treatments for oral biofilm-associated illnesses and MDR bacterial infections.

## Data Availability

The datasets generated and/or analyzed during the current study are available upon reasonable request from the corresponding author. “The National Center for Biotechnology Information [NCBI] received clinical isolates data of Klebsiella pneumoniae A11 and B. subtilis A33, which were assigned accession numbers PP995146 and PP995148, respectively.”
